# To the editor-an update on endovascular treatment of venogenic erectile dysfunction

**DOI:** 10.1186/s42155-022-00310-5

**Published:** 2022-06-29

**Authors:** Hanno Hoppe, Nicolas Diehm

**Affiliations:** 1 SwissIntervention Microtherapy Center, Kornhausstrasse 8, 3013 Bern, Switzerland; 2 University of Bern, Bern, Switzerland; 3 Vascular Institute Central Switzerland, Aarau, Switzerland

## To the editor

We refer to our case report „Venogenic erectile dysfunction: diagnosis on computed tomography cavernosography and endovascular treatment using an anterograde access via deep dorsal penile vein “as recently published in CVIR Endovascular (Hoppe and Diehm 2022).

### Supplementary procedural aspects

**Fig. 1:**
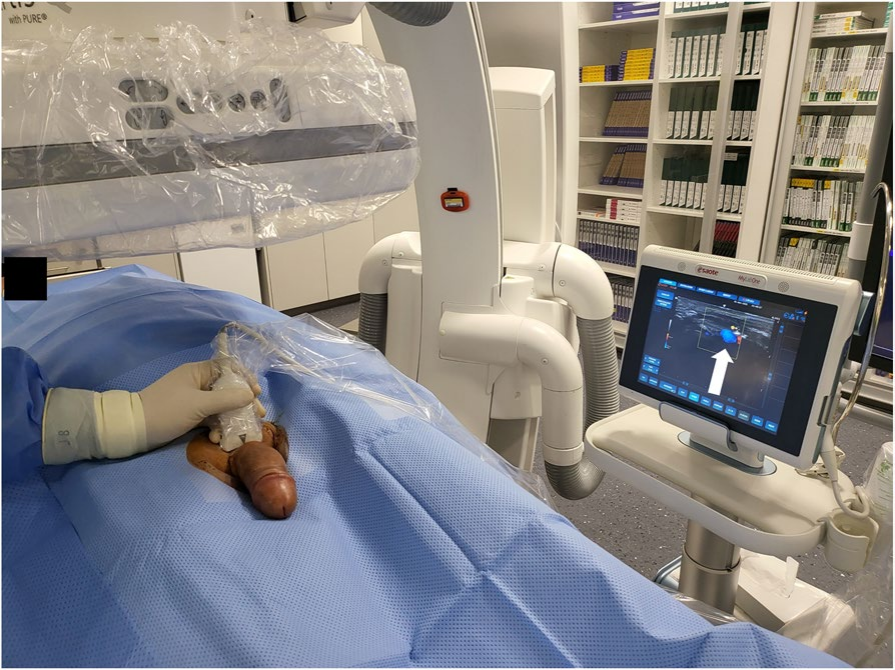
Angiosuite setup with fluoroscopy and ultrasound. This patient’s penis is sterilely prepped and draped. A 9 MHz ultrasound probe is also sterilely draped and color duplex is used to localize a proximal deep dorsal penile vein (arrow)

**Fig. 2:**
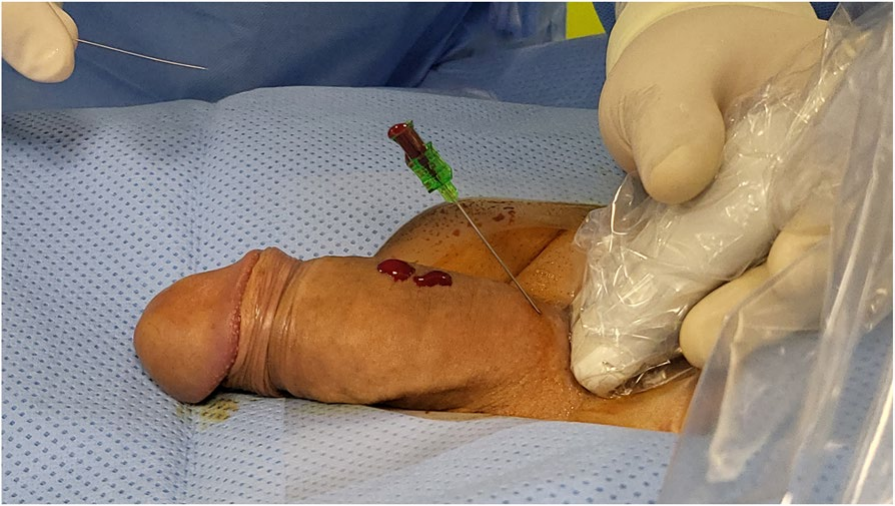
After subcutaneous administration of lidocaine 2% for local anesthesia and light sedation, ultrasound-guided puncture of a deep dorsal penile vein is performed using a 21-G micropuncture needle (Cook, U.S.A.). The needle is slowly advanced until blood returns from the needle hub (ref. corresponding movie file)

**Fig. 3:**
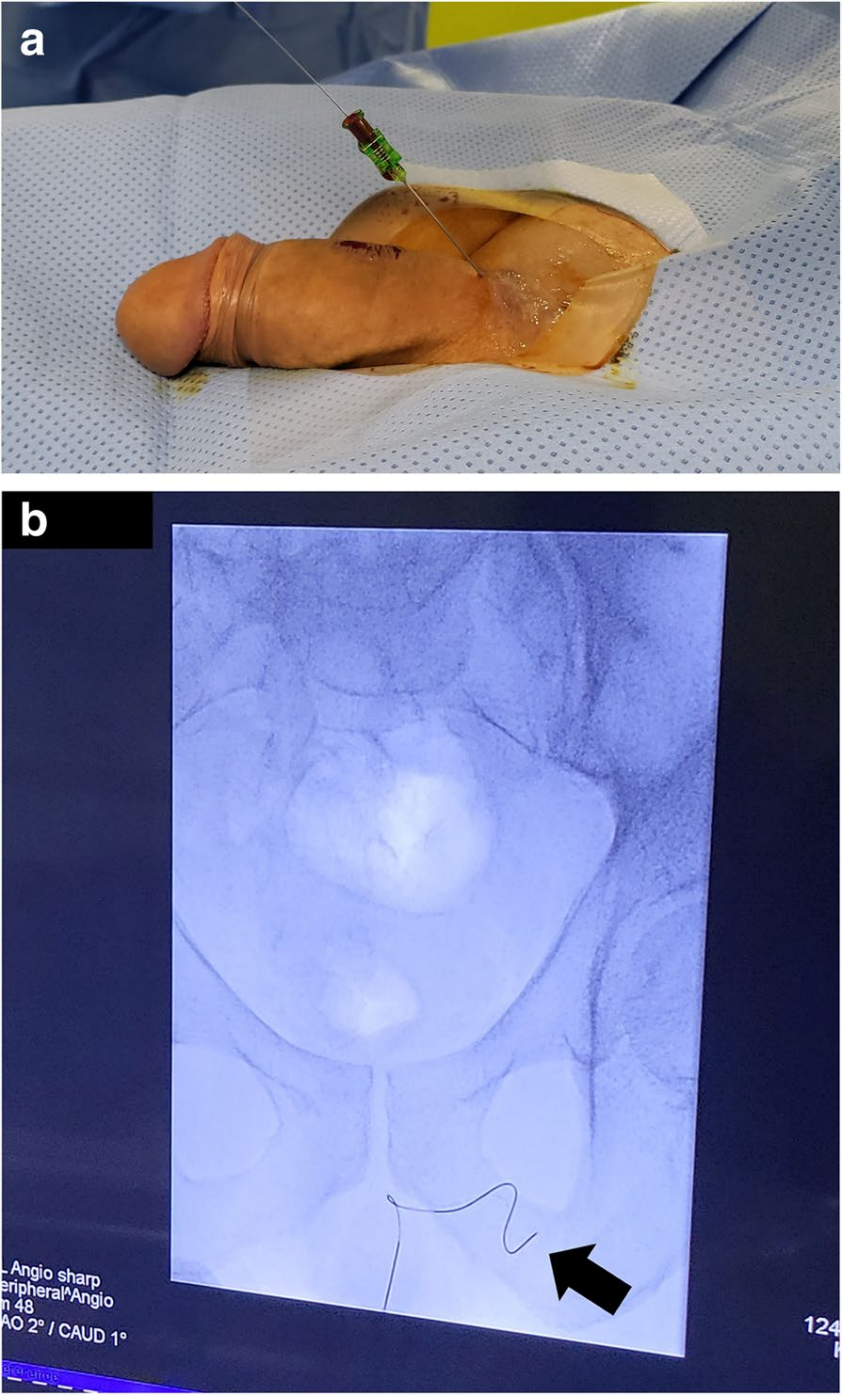
**A** An 0.018-inch guide wire is inserted into the needle hub and carefully advanced as long as there is no resistance. **B** Subsequently, position of the wire tip, in this case in the left internal pudendal vein (arrow), is checked under fluoroscopy

**Fig. 4:**
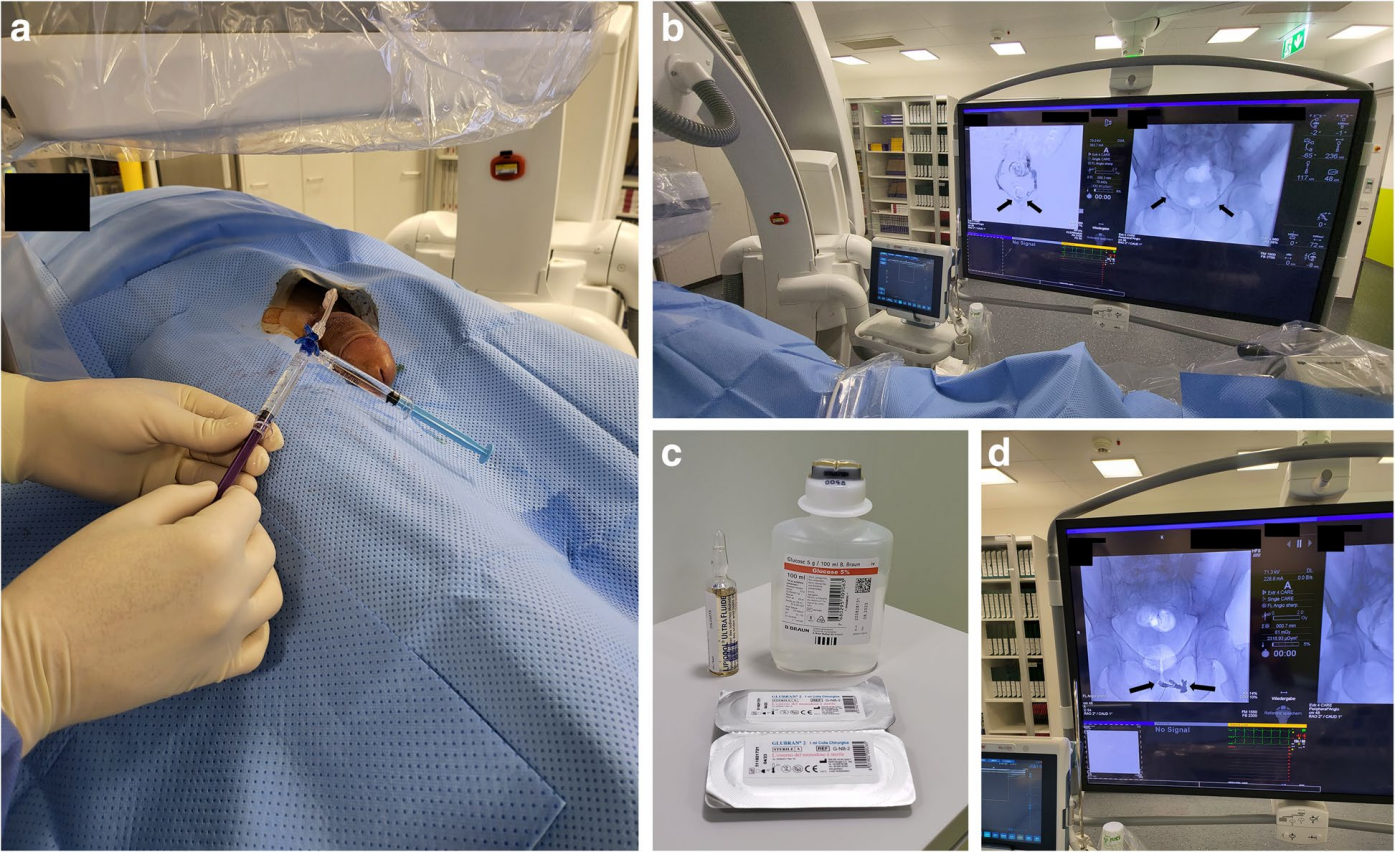
**A** A 3-F stiffened cannula out of a 4-F micropuncture set (Cook, U.S.A.) is inserted over the guide wire after the needle has been removed with its tip positioned intravenously in close proximity to the radix penis. Usually, a stiff dilator is more advantageous compared to a floppy one due to roughness of the penile fascia (Buck’s fascia). The introducer hub is connected to a 3-way stopcock with a short connecting tube (Discofix, Braun, Germany). **B** Venogram demonstrates venous leak (arrows) via periprostatic and internal pudendal veins. **C** Tubing is flushed with 5% glucose solution (light blue 3 cc syringe) and subsequently venous embolization is performed (violet 3 cc syringe) using a mix of N-butyl-2-cyanoacrylate glue (Glubran II, GEM, Italy) and ethiodized oil (Lipiodol, Guerbet, Switzerland), as exemplarily demonstrated. **D** Distribution of embolization material via periprostatic and internal pudendal veins (arrows)

With the intention to make this endovascular treatment more easily accessible to endovascular interventionalists, we compiled additional visual material straight from the angiosuite including a movie file to step-by-step demonstrate endovascular treatment of venogenic erectile dysfunction using an anterograde access via a deep dorsal penile vein (Figs. 1, 2, 3 and 4)

### Novel procedural aspects

As previously mentioned, we use a micropuncture set with a 21-G needle, an 0.018-inch guide wire and a stiffened cannula for ultrasound guided deep dorsal penile vein access. Use of a stiffened cannula appears to be more advantageous compared to a floppy cannula due to roughness of the penile fascia (Buck’s fascia). Recently we figured out that a stiff 3-F inner dilator is easier to introduce through the penile fascia into the deep dorsal vein without the 4-F outer catheter. Of interest, there is no relevant impairment of liquid embolic agents’ flow characteristics. This finding is confirmed by previous study results of Palacios et al. demonstrating that 3-F inner dilators are capable of achieving flow rates of at least 6 mL/sec (Palacios et al. 2009).

### Future perspective

However, despite of the promising result of this case report, more scientific evidence is needed regarding endovascular treatment of erectile dysfunction, especially in patients with venous leak. In the meantime, we have treated more than 50 patients for venogenic erectile dysfunction using an endovascular approach with antero-grade access via a deep dorsal penile vein and are currently working on a data analysis and publication of our results in the near future for further clarification.

## Supplementary Information

**Additional file 1. **

## Data Availability

All data generated or analysed during this study are included in this published article.

## Abbreviations

G: Gauge
F: French
